# Administration of encapsulated L-tryptophan improves duodenal starch digestion and increases gastrointestinal hormones secretions in beef cattle

**DOI:** 10.5713/ajas.19.0498

**Published:** 2019-11-01

**Authors:** Sang-Bum Lee, Kyung-Won Lee, Tao Wang, Jae-Sung Lee, U-Suk Jung, Jalil Ghassemi Nejad, Young-Kyoon Oh, Youl-Chang Baek, Kyoung Hoon Kim, Hong-Gu Lee

**Affiliations:** 1 Department of Animal Science and Technology, Sanghuh College of Life Sciences, Konkuk University, Seoul 05029, Korea; 2 Life Science Technology, Inc., Seoul 06134, Korea; 3 Department of Animal Nutrition and Feed Science, College of Animal Science and Technology, Jilin Agricultural University, Jilin 130118, China; 4 Team of an Educational Program for Specialists in Global Animal Science, Brain Korea 21 Plus Project, Sanghuh College of Life Sciences, Konkuk University, Seoul 05029, Korea; 5 Department of Nutrition and Physiology, National Institute of Animal Science, RDA, JeonJu 55365, Korea; 6 Graduate School of International Agricultural Technology, Pyeongchang Campus, Seoul National University, Pyeongchang 25354, Korea

**Keywords:** α-Amylase, Cholecystokinin, L-tryptophan, Monogastric, Polygastric Model, Protein

## Abstract

**Objective:**

This study investigated the effects of oral administration of rumen-protected L-tryptophan (RPL-T) on duodenal starch digestion and gastrointestinal hormones (GIH) secretions using Hanwoo beef steers as the animal models.

**Methods:**

Four steers (423±24 kg) fitted with ruminal and duodenal cannulas were employed in a crossover design replicated twice. Treatments were control (basal diet) and RPL-T (basal diet+191.1 mg/kg body weight [BW]) group. Blood and duodenal samples were collected to measure serum GIH levels and pancreatic α-amylase activity at day 0, 1, 3, and 5 (−30, 30, 90, 150, and 210 min) of the study. Samples from each segment of the gastrointestinal tract were collected via ruminal and duodenal cannulas and were used to determine soluble protein and the starch digestion rate at days 6 (−30, 180, 360, and 540 min) and 8 (−30, 90, 270, and 450 min) of the experiment.

**Results:**

No significant difference in ruminal pH, NH_3_-N, and total volatile fatty acid including the levels of acetate, propionate, butyrate, isobutyrate, valerate, isovalerate, and the acetate-to-propionate ratio was observed between groups (p>0.05). Crude protein uptake was higher and feces starch content was lower in RPL-T group than the control group (p<0.05). The D-glucose contents of feces in RPL-T group decreased at day 5 compared to those in the control group (p<0.05), however, no change was found at day 0, 1, or 3 compared to the control group (p>0.05). Serum cholecystokinin (CCK), melatonin, duodenal pancreatic α-amylase activity, and starch digestion were significantly higher in RPL-T group than the control group (p<0.05).

**Conclusion:**

Taken together, oral administration of RPL-T at the rate of 191.1 mg/kg BW consistently increased CCK concentration, pancreatic α-amylase activity in duodenal fluids, and starch digestion rate in the small intestine and thus found to be beneficial.

## INTRODUCTION

L-tryptophan (L-T) is involved in the production of serotonin [1,2], melatonin (MEL) [3,4], and a precursor of nicotinic acid [5]. Precedent studies in monogastric animals (e.g., humans, pigs, dogs, rats, mice, and chickens) elucidated the pivotal biological role of tryptophan (TRP) in association with different metabolic processes [6] including increased MEL in rainbow trout [7] that is also associated with stressed animals [3]. In humans, infusion of L-T has been shown to increase prolactin and growth hormone secretions [8]. Moreover, in mammalian, the protective roles of MEL and TRP on increase of gastric mucosa and stimulation of cholecystokinin (CCK) and pancreatic exocrine function via enteropancreatic reflex mechanism has been reported [4]. However, little is known about the possible similar effects in ruminants as polygastric animal models.

Given that undigested starch in the rumen of polygastric animals passes into the lower digestive organs, a small amount of α-amylase is secreted into the small intestine with extremely low activity where only 35% to 60% of starch is digested [9]. The remaining 35% to 50% of the starch is excreted in undigested form through feces [10]. In other words, starch digestion in the small intestines of ruminants can be modulated by the environment inside of the small intestine. It is while a similar phenomenon was previously reported in broiler chicks [11] and weaned piglets [12]. Therefore, any attempts to improve α-amylase activity in duodenal fluids could help starch digestion, which is also targeted in the present study.

The most recent study in our laboratory [13] demonstrated that the intravenous administration of L-T (57.8 mg L-T/kg body weight [BW]) increased the secretion of MEL in growing beef steers. From these results, and due to the rate of absorption and disappearance of L-T (~72%) in gastro-intestinal (GI) tract, we hypothesized that using ~200 mg/kg BW of RPLT may lead to similar results if provided orally. Additionally, there are GI related hormones that stimulate the rate of feed digestion including CCK, MEL, and secretin that are associated with L-T. As demonstrated by Leja-Szpak et al [14] the effects of the administration of L-T on MEL synthesis in mice resulted in higher MEL levels. Both L-T and MEL are closely related to CCK, an endocrine hormone that can eventually increase the synthesis of pancreatic α-amylase. In studies on ruminants, administration of L-T to the jugular vein of dairy cows reported increasing the secretion of serotonin [14]. Additionally, evidences showed that an increase in MEL and ghrelin by L-T stimulated the secretion of CCK and α-amylase in monogastric animals [14,15]. However, it is yet to prove the role of L-T in association with starch degradation in polygastric animals. Therefore, this study was undertaken to determine if similar endocrine and digestive effects would be elicited by oral administration of RPL-T empirically using beef cattle as experimental animal models. We elected not to observe changes in ruminal characteristics due to using encapsulated (rumen-protected) L-T; and thus to test this hypothesis and in addition to measure the main parameters, we analysed the rumen fluid characteristics in this study to see if L-T in the encapsulated form can successfully bypass through the rumen without degradation.

## MATERIALS AND METHODS

### Animals and treatment preparation

All experimental procedures were in accordance with the “Guidelines for Care and Use of Experimental Animals” of Pusan National University (approval no.: PNU-2010-000152). Four Korean native steers (ave. BW = 423±24 kg) and cannulated in their rumen and duodenum were employed. Animals were kept in the individual cattle sheds with concrete floors and fence of 3×3.2 m under illumination and heat control (16 h of lights-on, 8 h of lights-out at 23°C). They were provided free access to water and salt blocks. Basic feed was composed of rice straw (31.3%) and growing-fattening feed (68.7%). Diet were formulated by Grobig DC (vitamin A, 2,650,000 IU; vitamin D_3_, 530,000 IU; vitamin E, 1,050 IU; nicotinic acid, 10,000 mg; Fe, 13,200 mg; Mn, 4,400 mg; Zn, 4,400 mg; copper, 2,200 mg; iodine, 440 mg; cobalt, 440 mg) and were composed of dry matter (DM) = 93.5, starch = 48.8, curde protein (CP) = 14.1 (Supplementary Table S1-1) based on recommendations by National Institute of Animal Science [16]. The BW was measured prior to each period. Animals were fed twice daily at 0800 and 1700 h for 11 days before treatment. Metabolizable energy uptake was controlled to 1.2-times more than that required for Korean native steers [16] in order to ensure that each animal receiving enough energy uptake. Chrome oxide (Cr_2_O_3_) was added to the growing-fattening feed at 0.2% as a marker to measure the contents of digesta. The RPL-T (42% L-T, Beijing Feeding Feed Science Technology CO., Beijing, China) was used in this study. RPL-T is a mixture of granules (42% L-T, 37.7% fat, and 20.3% carbohydrates) wrapped by resin made of complex organic acids and their derivatives. The amount of RPL-T (191.1 mg/kg BW) used in this study was calculated based on the dose used in the most recent study [13] in our laboratory to define the effective dosages (57.8 mg/kg BW) and the average absorption rate (72%) of L-T in the small intestine of ruminants [17]. Steers were randomly assigned to each group in a crossover design, and the experiment was repeated twice. Animals were allowed to adapt to the basic feed for 11 days before treatment. After the adaptation period, two different diets were used: i) basal diet (31.3% of rice straw + 68.7% of concentrate) + RPL-T (encompassing 42% L-T), giving 191.1 mg/kg BW (treatment group); ii) basal diet + 58% of diluting agent, giving 191.1 mg/kg BW (control group). Steers were fed for 1 to 8 days per treatment period with 4 days of rest between treatment periods for a total of 20 days with two feeding treatment periods. The amount of diet for the treatment was determined based on individual BW measured 1 day prior to treatment administration.

### Sampling

#### Diet sampling

Daily feed uptake was recorded in this experiment. Samples of the diet were collected for 6 to 8 days of beginning, mid and end of experiment. All samples were ground to 1 mm in size using a Wiley mill (Thomas Scientific, Model 4, Swedesboro, NJ, USA) and dried for the following proximate analyses.

#### Serum sampling

Blood samples were harvested from the jugular vein in evacuated tubes with no additives to obtain serum. Times of the sample collections were at −30 min (0830 h), 30 min (0930 h), 90 min (1030 h), 150 min (1130 h), and 210 min (1230 h) on day 0 (before treatment), day 1, day 3, and day 5. After blood collection, serum was obtained by centrifugation (1,200×g for 20 min, 4°C) and then placed into storage tubes and stored at −20°C until further analysis.

#### Duodenum fluids sampling

Duodenum fluids (250 mL) were sampled through a duodenum cannula to analyze the rate of starch digestion in each section of the intestine at −30 min (0830 h), 180 min (1200 h), 360 min (1500 h), and 540 min (1800 h) on day 6, and at −30 min (0830 h), 90 min (1030 h), 270 min (1330 h), and 450 min (1630 h) on day 8. These fluids were lyophilized (Programmable Freeze dryer, Ilshin lab Co., Ltd., Seoul, Korea) and ground to 1 mm in size using a Disc Mill (Model BM-D 100, McCoy Corporation, San Marcos, TX, USA). Samples were mixed together for the analysis of DM, CP, ash, and starch [18]. A 50 mL sample of duodenal fluid was sampled via the cannula for the analysis of α-amylase activity at −30 min (0830 h), 30 min (0930 h), 90 min (1030 h), 150 min (1130 h), and 210 min (1230 h) on days 0, 1, 3, and 5 of each period. Samples were frozen at −80°C before analysis.

#### Rumen fluids sampling

A 300 mL ruminal fluid was collected for the analysis of starch digestion at each section of the intestine at −30 min (0830 h), 60 min (1000 h), 180 min (1200 h), and 300 min (1400 h) for each treatment on day 6 and day 8. The fluids were filtered with a four-layer gauze immediately after sampling. The pH was measured using Seveneasy pH meters (Mettler-Toledo AG 8603, Schweraenbach, Switzerland) followed by a preconditioning process to analyze ammonia and volatile fatty acids (VFA). Samples were frozen at −80°C before analysis.

#### Feces sampling

Approximately 300 g of feces were obtained from the rectum using a recto-vaginal technique at the same collection time that duodenal fluids were taken, and stored at −80°C. Samples were dried and ground to 1 mm in size for the analysis of the DM, CP, ash, and starch using AOAC [18]. Chromium oxide (Cr_2_O_3_) was used to track digestion in a different section of GI tract.

### Analyses

#### Ruminal characteristics (NH_3_-N and VFA)

The concentration of ammonia was analyzed using the method by Chaney and Marbach [19]. Samples were preconditioned by adding 10 μL of HgCl_2_ to 1 mL of ruminal fluids in a 1.5 mL micro tube. Samples were mixed to deactivate microorganisms in the rumen. Samples were stored in a deep freezer (−80°C) until analysis. During the assays, samples were thawed at room temperature and centrifuged (13,500×g, 5 min) to obtain 20 μL of supernatant. Supernatant, distilled water, and ammonia standard solutions (2.5, 5, 10, 20, and 40 mg NH_3_-N/100 mL) were added to three sets of 20 mL tubes. One milliliter of phenol color reagent (distilled water 1 L; phenol [C_6_H_5_OH] 50 g; sodium nitroferricyanide [Na_2_(Fe[CN]_5_NO)·2H_2_O]; alkali-hypochlorite reagent [distilled water 1 L, sodium hydroxide (NaOH) 25 g]; and sodium hydrochloride [4% to 6% NaOCl] 16.8 mL) was added to each tube, mixed, sealed with a butyl rubber stopper, and incubated at 37°C in a constant-temperature water bath for 15 min. After incubation, 8 mL of distilled water was added to measure the absorbance (optical density) at 630 nm using a spectrophotometer (Bio-Rad, US/benchmark plus, Tokyo, Japan). Ammonia concentrations in the samples were determined using a standard calibration curve. The VFAs in the rumen was measured using the method described by Erwin et al [20]. Samples were preconditioned by adding 10 μL of HgCl_2_ to 1 mL of rumen fluids in a 1.5 mL micro tube. Samples were mixed to deactivate microorganisms in rumen. After that, 200 μL of H_3_PO_4_ was added to remove proteins from microorganisms, and 40 μL of pivalic acid was added as an internal standard for gas chromatography (GC). The solution was mixed and stored in a deep freezer (−80°C) until analysis. All analyses were repeated at least three times. During assays, samples were thawed at room temperature and centrifuged (13,500×g, 5 min) to obtain the supernatant for GC analysis (VARIAN model CP-3800, Walnut Creek, CA, USA). The VFAs standard solution was made by mixing 100 mL distilled water, with acetate 350 μL, propionate 150 μL, isobutyrate 50 μL, butyrate 100 μL, isovalerate 50 μL, valerate 50 μL, and 5 mL of standard solution (200 μL of pivalic acid and 1.05 mL of distilled water). One milliliter of this solution was used as a VFA standard. The contents of VFA in the sample were determined using a standard calibration curve.

#### Immunoassay for establishing and analyzing GIH (ghrelin, secretin, and CCK-8) and MEL in bovine serum

Serum concentrations of ghrelin, secretin, and CCK-8 were measured by enzyme immunoassay using secondary antibodies for ghrelin (EK-031-30), secretin (EK-067-05), and CCK-8 (EK-069-04) (Phoenix Pharmaceuticals, Burlingame, CA, USA) with a human MEL ELISA Kit (RE54021) (IBL, Hamberg, Germany). Serum was prepared for analyses and to test the cross-reactivity of standards in the kit using the method described by Lee et al [6].

#### Determination of α-amylase activity in duodenal fluid of the exocrine pancreas

α-Amylase activity in duodenal fluid secreted from the exocrine glands of the pancreas was determined using the EnzyChrom α-amylase Assay Kit (ECAM-100, BioAssay Systems, Hayward, CA, USA).

#### Analysis of starch digestibility in gastrointestinal fluids

To analyze starch digestibility in each section of the intestine, the Anthrone Method [21] using D-glucose standard solution and the method described by McCready et al [22] were used. In brief, frozen intestinal fluid was thawed at room temperature and ground to less than 0.5 mm in an icebox using a homogenizer. The homogenate was filtered through a 2-layer gauze (sieve less than 0.5 mm). Five-milliliters of anthrone solution (0.1 g of anthrone dissolved in 100 mL of the solution made by diluting 760 mL of sulfuric acid to distilled water to make 1 L) was added to 1 mL of supernatant to assess color change. After centrifugation, supernatants were removed and the precipitates were dried at 40°C to 50°C for 60 min to completely remove ethanol. Care was taken to keep the temperature below 60°C in order to prevent the degradation of glucose and protein. After drying, 5 mL of distilled water was added and mixed well followed by the addition of 6.5 mL of 52% perchloric acid reagent. Next, supernatants were filtered and moved to a 100 mL volumetric flask. The waste residue of the sample was filtered with distilled water to a final volume of 100 mL, which was the last step of preconditioning. One milliliter of the sample solution and different concentrations of standard solutions (25, 50, 100, 150, and 200 μg/mL) were added to 20 mL tubes containing 5 mL of anthrone reagent. After mixing well, the solution was incubated at 100°C in a constant-temperature water bath for 12 to 13 min, after which it was cooled down to room temperature immediately after color development to measure the absorbance at 630 nm using a spectrophotometer. All samples were then diluted as appropriate for subsequent measurements. The rate of starch digestion was converted to starch contents, calculated from the D-glucose contents of samples.

### Statistics

Data of serum MEL, GIH, duodenum α-amylase, D-glucose, ruminal characteristics, apparent rates of crude protein disappearance, and apparent rates of starch disappearance were analyzed using Student’s paired t-test. All statistical analyses were performed using SPSS software package (SPSS, Chicago, IL, USA). Statistical significance was considered when the p-value was less than 0.05 or 0.01.

## RESULTS

No significant differences (p>0.05) were observed in ruminal pH, NH_3_-N, and total VFA including the levels of acetate, propionate, butyrate, isobutyrate, valerate, isovalerate, and the acetate-to-propionate ratio between two groups (Table 1). The rates of CP flow and digestion in each section of the intestine according to the dietary supply of RPL-T are summarized in Table 2. Crude protein uptake by the RPL-T group was 8.6 g/d higher (p<0.0001) than that by the control group. However, no significant differences (p>0.05) were found in the flow of CPs or in the amount of digesta flowing into the small intestine between the RPL-T group and the control.

The flow rate and digestion rate of starch in each section of the intestine according to the dietary supply of RPL-T are summarized in Table 3. Starch content in the feces of the RPL-T group was significantly lower (p<0.05) than that in the control group, although the influx of starch to the small intestine was significantly higher (p<0.05) than that in the control group. Both the rate and amount of starch digestion increased (p<0.05) in total tract following by RPL-T administration.

**Figure 1:**
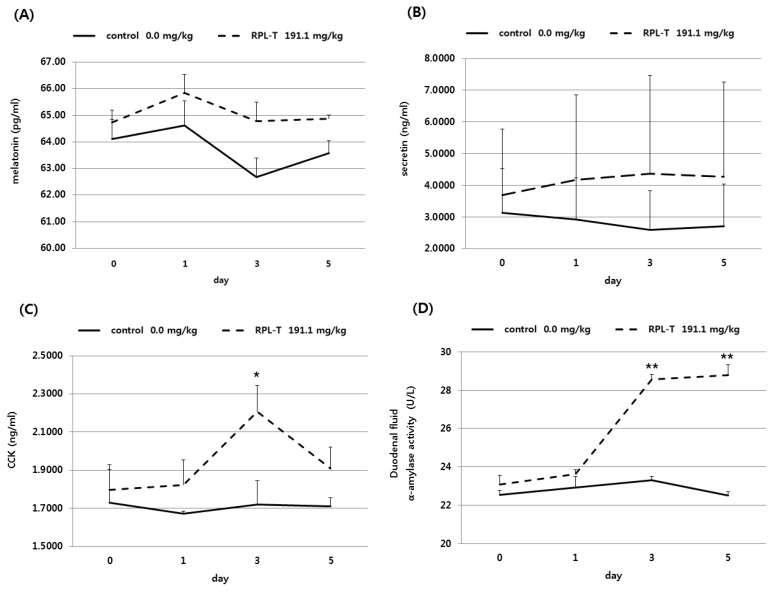
Changes in the levels of serum melatonin (A), secretin (B), CCK (C), and duodenum α-amylase (D) in Korean native steers fed RPL-T. CCK, cholecystokinin; RPL-T, rumen-protected L-tryptophan. Mean±standard error, * p<0.05, ** p<0.01 (Student’s paired *t*-test, control vs RPL-T at 191.1 mg/kg body weight). — Control group, - - - RPL-T group.

The effects of oral RPL-T administration on serum MEL synthesis, intestinal hormones (secretin and CCK), and α-amylase activity of pancreatic exocrine duodenal fluids are shown in Figure 1 and Table 4. Serum MEL level increased (p<0.05) following oral administration of RPL-T compared to that in the control group, however, there was no significant difference (p>0.05) among all periods (i.e., day 0, 1, 3, and 5). An increase (p<0.05) in MEL was observed in the RPL-T group compared to that in the control group (Table 4). There was no difference (p>0.05) in CCK concentration in the RPL-T group until day 1, with a rapid and significant increase (p< 0.05) at day 3 compared to the control group (Figure 1). Similarly, there was no significant increase in α-amylase activity in the duodenum digesta of the RPL-T group until day 1. However, it was significantly increased (p<0.05) at day 3 and 5 compared to that in the control.

**Figure 2:**
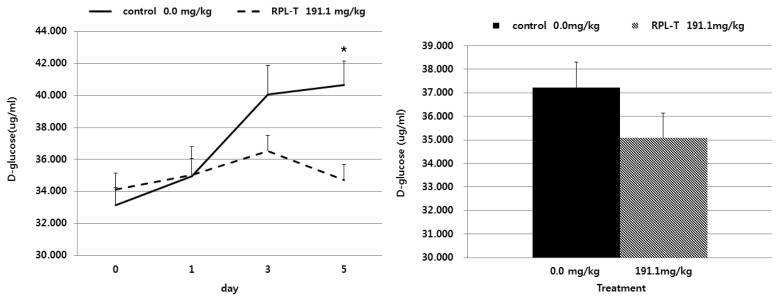
Effects of oral RPL-T on D-glucose content in feces of Korean native steers. RPL-T, rumen-protected L-tryptophan. Mean±standard error, * p<0.05 (Student’s paired *t*-test, control vs RPL-T at 191.1 mg/kg body weight). — Control group, - - - RPL-T group.

Changes in the D-glucose content in feces followed by oral administration of RPL-T are shown in Figure 2. The D-glucose contents in the feces of the group orally administered RPL-T were significantly decreased (p<0.05) at day 5 compared to those in the control group. However, no change (p>0.05) was found at day 0, 1, or 3 compared to the control. The rate of starch digestion was also significantly increased (p<0.05) in the oral RPL-T group compared to the control group (Table 4).

## DISCUSSION

The reason why we measured ruminal fluid characteristics in the animals including pH, NH_3_-N, and VFAs was to find out if the encapsulated L-T could be successfully bypassed via rumen. Although bypassing 100% of encapsulated L-T is not assumed. However, as shown in Table 1, there was no difference in ruminal fermentation based on the dietary supply of RPL-T between two groups, including for ruminal pH, total VFA, ruminal ammonia concentration, and other factors. This phenomenon may be due to feeding the encapsulating L-T that helped to bypass L-T through rumen. In other words, the rate of retained L-T in the rumen was not high enough to affect rumen fluid characteristics. In addition, Ludden et al [23] reported that an increase in the protein supply to ruminants could lead to an increase in branched-chain VFAs. However, dietary supply of RPL-T showed no effect on rumen fermentation in the present study. A previous study reported that L-T could be degraded by microorganisms in the rumen before entering the small intestine for absorption in ruminants [5]. However, this is unlikely during ruminal fermentation because L-T was protected by fats and carbohydrates in RPL-T product in order to prevent degradation by microorganisms in the rumen. As we fed rumen protected L-T, no changes in ruminal fermentation could be expected. The reason why we measured ruminal fermentation characteristics was that we wanted to make sure the product remains intact in the rumen. Conforming with our expectation, rumen fluid characteristics showed no alteration following oral administration of RPL-T indicating successful bypassing of the product through the rumen and that it escaped degradation.

Table 2 shows the flow and digestion rate of CP at each section of the intestine according to the dietary supply of RPL-T. Although the uptake of CP was higher in the RPL-T group, RPL-T had no effect on the contents or the digestion rate of CP in each section of the intestine. Titgemeyer et al [24] noted that the dietary supply of ruminally undegradable proteins could increase the influx of N and amino acids into the small intestines. In addition, Goedeken et al [25] reported increased influx of N and amino acids into the small intestine, including increased nitrogen availability and growth. However, an excessive supply of rumen-undegradable protein (RUP) could decrease the efficiency of amino acid flow into the small intestine and protein synthesis [26]. High levels of RUP can limit the protein synthesis of microorganisms, leading to decreased concentrations of the final products of ruminal degradation, such as NH_3_-N, amino acids, or branched chain VFAs [27]. However, the amount of RUP in the present study was not excessive because the influx of RPL-T into the small intestine was decreased. RPL-T had no effect on the environment of the rumen or on ruminal microorganisms. It only affected the protein being absorbed or used in the small intestine due to the fact that it was encapsulated to be protected in the rumen.

The D-glucose contents in feces decreased over the time following by oral administration of RPL-T (Figure 2), with a significant decrease on day 5 compared to the control group. These results may be due to the direct stimulation of I-cells in the mucosa by RPL-T entering to the duodenum, and through the indirect stimulation of endogenous MEL synthesized from L-T [14]. Both I-cells and endogenous MEL may have induced the secretion of intestinal CCK, which eventually enhanced the activity of pancreatic α-amylase and decreased fecal D-glucose contents [14]. When considering the flow and digestion rate of starch at each section of the intestine according to the dietary supply of RPL-T (Table 3), a significant decrease in starch contents was observed in the RPL-T group compared to the control group as shown in the present study. Small intestinal starch assimilation is limited in ruminants [28]. Richards et al [28] showed that comparing with infusing starch alone, infusing protein with starch into the small intestine enhanced small intestinal starch disappearance. They also reported that the products of feed protein breakdown, such as peptides or amino acids, entering to the small intestine could modulate starch bioavailability in the small intestine and RUP, thus enhancing their bioavailability [28]. In particular, Choi et al [29] noted that an additional dietary supply of soluble proteins can lead to an influx of amino acids and peptides into the lower intestines, thus affecting the practical productivity of ruminants such as growth, milk production, and other factors. Therefore, oral administration of RPL-T affected MEL concentration and CCK secretion (Table 4) associated with starch digestion by enhancing the absorption of CPs (L-T) in the rumen and small intestine and eventually enhancing the rate of starch digestion in the small intestine.

A significant increase in serum MEL, CCK concentration, and pancreatic α-amylase activity in the duodenum were observed in the RPL-T group compared to the control group (Figure 1; Table 4). This is consistent with the findings of Barry et al [30], who reported that increased absorption of amino acids can modulate the hormonal status. Oral administration of L-T could reportedly increase plasma concentration of MEL in humans [1], chickens, mice [6], and cows [31]. In this study, and in agreement with the above studies, the aforementioned results in human and the other animal species were confirmed. Furthermore, induction of endogenous MEL by L-T strongly stimulated the secretion of pancreatic proteins [14]. Leja-Szpak et al [14] reported that administration of the essential amino acid L-T to mice can affect the synthesis of the pineal hormone MEL. Therefore, since both L-T and MEL are closely related to the intestinal hormone CCK [4], the production of pancreatic α-amylase was increased. In general, the secretion of CCK is stimulated by the degradation of chain peptides and small molecules that enter the duodenum [32]. Secretion of CCK is related to the uptake of chained amino acids [33]. Additionally, in our study, there was no significant difference in the daily CCK between the L-T and control group until day 1. CCK concentrations increased significantly at day 3 in the L-T group but decreased at day 5. This could be related to the amount of endogenous MEL synthesized from L-T [1]. The synthesis of MEL may not be continuously maintained at high level. Similarly, oral RPL-T had no significant effect on the daily CCK concentration compared to the control group until day 1. However, CCK activity increased significantly at day 3 and 5. Protein hydrolysates can flow into the small intestine and instantly stimulate the secretion of CCK by isolated mucosal cells [34] or STC-1 cells of neurosecretion [35]. Secreted CCK can stimulate acinar cells in the pancreas by the afferent pathway of the pneumogastric nerves to stimulate the secretion of pancreatic fluids [36]. However, more studies are needed to determine how CCK stimulates CCK receptors of acinar cells in the pancreas and to confirm this result.

Serum secretin in the oral RPL-T group was higher than that in the control group. However, variability among individual responses made it difficult to draw a conclusion in this regard. Secretin has been extensively investigated for its peripheral effects on digestion [37]. Postprandial secretion of pancreatic fluids largely depends on the activation of cholinergic postganglionic neurons of the pancreas by valgus reflex of intestinal hormones, such as secretin and CCK [38]. Rausch et al [39] reported an increase in the synthesis of pancreatic proteins by a maximum of 90% compared to the control group when secretin was infused for 6 and 12 h into the acinar cells in mice. The aforementioned results complied with the recent rat study [38]. Manso et al [40] also showed that the total amount of protein synthesized from the pancreas and amylase secretion increased when secretin administered to mice with acute pancreatitis. However, little is known about the relationship between L-T and the secretion of secretin. The relationship between oral administration of RPL-T and the secretion of secretin remained unclear in the present study and thus need further investigations.

## CONCLUSION

Taken together, oral administration of RPL-T at the rate of 191.1 mg/kg BW is advisable due to its effects on consistently increasing CCK concentration, pancreatic α-amylase activity in duodenal fluids, and starch digestion rate in the small intestine.

## Supplementary Data



## Figures and Tables

**Table 1:** Ruminal characteristics in Korean native steers fed basal diet and rumen-protected L-tryptophan (RPL-T)

Items	Treatment1)		SEM	p-value2)

	Control	RPL-T		
pH	6.34	6.28	0.09	ns
Total VFA, mmol	82.80	77.40	6.84	ns
VFA concentration, mol/100 mL				
Acetate, mol (%)	58.00	55.40	1.06	ns
Propionate, mol (%)	20.90	23.10	0.74	ns
Butyrate, mol (%)	14.70	15.40	0.42	ns
Isobutyrate, mol (%)	1.38	1.23	0.12	ns
Valerate, mol (%)	1.74	1.87	0.07	ns
Isovalerate, mol (%)	3.26	3.04	0.28	ns
Acetate:propionate	2.85	2.57	0.13	ns
NH_3_-N, mg/L	123.00	117.60	16.54	ns

RPL-T, rumen-protected L-tryptophan; SEM, standard error of the mean; VFA, volatile fatty acids; BW, body weight.

1) Control, concentrate 7 kg/d + rice straw 1 kg/d + excipient average 110.8 mg/kg BW; RPL-T, concentrate 7 kg/d +rice straw 1 kg/d + RPL-T 191.1 mg/kg BW.

2) p-values were calculated by paired t-test (ns = non-significant).

**Table 2:** Apparent crude protein loss in each segment of the gastrointestinal tract in Korean native steers fed basal diet and RPL-T

Items	Treatment1)		SEM	p-value2)

	Control	RPL-T		
Crude protein intake (g/d)	972.4^b^	981.0^a^	1.62	<0.0001
Duodenal flow (g/d)	480.9	477.3	6.15	ns
Fecal flow (g/d)	193.4	184.4	4.86	ns
Disappearance (g/d)				
Rumen	491.5	503.7	6.52	ns
Intestine	287.5	292.9	7.01	ns
Total tract	779.0	796.6	5.68	ns
Disappearance (%)				
Rumen, of intake	50.5	51.3	0.64	ns
Intestine, of flow	59.8	61.3	1.05	ns
Total tract, of intake	80.1	81.2	0.51	ns

RPL-T, rumen-protected L-tryptophan; SEM, standard error of the mean; BW, body weight.

1) Control, concentrate 7 kg/d + rice straw 1 kg/d + excipient average 110.8 mg/kg BW; RPL-T, concentrate 7 kg/d + rice straw 1 kg/d + RPL-T 191.1 mg/kg BW.

2) p-values were calculated by paired *t*-test (* p<0.05 and ns = non-significant).

a,b Values within a row with different superscripts differ significantly at p<0.05.

**Table 3:** Apparent starch loss in each segment of the gastrointestinal tract in Korean native steers fed basal diet and RPL-T

Items	Treatment1)		SEM	p-value2)

	Control	RPL-T		
Starch intake (g/d)	3,196.0	3,196.0	0.00	
Duodenal flow (g/d)	797.7	882.3	117.90	ns
Fecal flow (g/d)	224.4^a^	164.2^b^	15.72	^*^
Disappearance (g/d)				
Rumen	2,398.7	2,314.1	117.90	ns
Intestine	573.3	718.1	119.88	ns
Total tract	2,972.0^b^	3,032.2^a^	15.72	^*^
Disappearance (%)				
Rumen, of intake	75.0	72.4	3.69	ns
Intestine, of flow	67.8	78.9	4.39	ns
Total tract, of intake	93.0^b^	94.9^a^	0.49	^*^

RPL-T, rumen-protected L-tryptophan; SEM, standard error of the mean; BW, body weight.

1) Control, concentrate 7 kg/d + rice straw 1 kg/d + excipient average 110.8 mg/kg BW; RPL-T, concentrate 7 kg/d + rice straw 1 kg/d + RPL-T 191.1 mg/kg BW.

2) p-values were calculated by paired t-test.

a,b Values within a row with different superscripts differ significantly at p<0.05.

**Table 4:** Effect of oral administration of rumen-protected L-tryptophan for 5 days on levels of serum MEL, GIH, and duodenum α-amylase in Korean native steers

Items	Control	RPL-T
Hormones		
Melatonin (pg/mL)	63.75±0.413	65.06±0.263*
Cholecystokinin (ng/mL)	1.71±0.054	1.93±0.069*
Secretin (ng/mL)	2.90±0.726	4.13±1.368
Enzyme		
α-Amylase activity (U/L)	22.81±0.174	26.01±0.712**

MEL, melatonin; GIH, gastrointestinal hormones; RPL-T, rumen-protected L-tryptophan; BW, body weight.

* p<0.05,

** p<0.01 (Student’s paired t-test, control vs RPL-T 191.1 mg/kg BW).
