# Supplementary material for: Administration of encapsulated L-tryptophan improves duodenal starch digestion and increases gastrointestinal hormones secretions in beef cattle
Source: Asian-Australas J Anim Sci. 2019 Nov 1;33(1):91–9. doi: 10.5713/ajas.19.0498 (PMC6946987; doi:10.5713/ajas.19.0498)
Supplement: Supplementary file 1 [file ajas-19-0498-suppl.pdf]

Supplementary Table

Table 1-1. Ingredient and chemical composition of basal diet, rice straw and RPL-T

Item	Basal diet	Rice straw	RPL-T
Ingredient (%, of air dry basis)			
Corn (Coarse cracked)	47.80		
Wheat bran	41.00		
Soybean meal	5.00		
Rapeseed meal	2.00		
Molasses	2.00		
Limestone	1.50		
Salt	0.40		
Grobig DC[1]	0.20		
Lasalocid[2]	0.10		
Total	100.00		
Chemical composition[3] (%, of DM)			
DM	93.48	92.21	98.85
Starch	48.85	-	-
CP	14.06	5.70	35.06
Ash	0.35	13.47	5.29
EE	-	-	50.81

[1] Grobig DC provided following nutrients per kg of diet : Vitamin A : 2,650,000 IU, Vitamin D_3 : 530,000 IU, Vitamin E : 1,050 IU, Nicotinic acid : 10,000 mg, Fe : 13,200 mg, Mn : 4,400 mg, Zn : 4,400 mg, Copper : 2,200 mg, Iodine : 440 mg, Cobalt : 440mg

[2] A type of innophore

[3] DM, dry matter; CP, crude protein, EE, ether extract.
